# STING agonist inflames the cervical cancer immune microenvironment and overcomes anti-PD-1 therapy resistance

**DOI:** 10.3389/fimmu.2024.1342647

**Published:** 2024-03-14

**Authors:** Tianye Li, Weijiang Zhang, Mengke Niu, Yuze Wu, Xinyue Deng, Jianwei Zhou

**Affiliations:** ^1^ Department of Gynecology, The Second Affiliated Hospital, Zhejiang University School of Medicine, Zhejiang University, Hangzhou, China; ^2^ Department of Oncology, Tongji Hospital of Tongji Medical College, Huazhong University of Science and Technology, Wuhan, China; ^3^ Otolaryngology & Head and Neck Center, Cancer Center, Department of Head and Neck Surgery, Zhejiang Provincial People’s Hospital (Affiliated People’s Hospital), Hangzhou Medical College, Hangzhou, Zhejiang, China; ^4^ Zhejiang Provincial Clinical Research Center for Obstetrics and Gynecology, Hangzhou, China

**Keywords:** cervical cancer, tumor microenvironment, STING agonist, anti-PD-1 treatment, single-cell RNA-seq

## Abstract

**Background:**

Cervical cancer poses a significant global threat to women’s health. However, current therapeutic interventions, such as radiotherapy, chemotherapy, surgical resection, and immune checkpoint inhibitors, face limitations in the advanced stages of the disease. Given the immunosuppressive microenvironment in cervical cancer, it is imperative to explore novel perspectives. In this regard, STING agonists have emerged as promising candidates.

**Methods:**

The expression profiles and clinicopathological data were obtained from The Cancer Genome Atlas (TCGA) and Gene Expression Omnibus (GEO) datasets. Prognostic analysis of STING downstream genes (CCL5, CXCL9, CXCL10) and immune infiltration analysis were conducted using Kaplan-Meier Plotter, ESTIMATE, and *deconvo_CIBERSOR*. Single-cell RNA-seq (scRNA-seq) analysis was conducted to evaluate the potential of MSA-2 in cervical cancer treatment employing SingleR, chi-squared test, and Gene Set Enrichment Analysis (GSEA). Cellular interaction analysis utilized the CellChat package to assess the potentiation of cellular interaction following MSA-2 administration. Murine tumor models involving U14 and TC-1, were conducted, and the IF of tissue was subsequently conducted to assess the tumor microenvironment status after treatment.

**Results:**

Prognosis in cervical cancer correlated with elevated expression of STING downstream genes, indicating prolonged survival and reduced recurrence. These genes positively correlated with immune infiltration, influencing stromal scores, immune scores, and estimate scores. Specific immune cell populations, including CD8^+^ T cells, M1-type macrophages, NK cells, and T follicular helper cells, were associated with STING downstream genes. scRNA-seq in a classic immune-excluded model revealed that MSA-2 exerts priming and activating functions on vital components within TME, and intensifies their intercellular communications. The *in vivo* assay ultimately demonstrated that MSA-2, either as a standalone treatment or in combination with anti-PD-1, effectively suppressed the growth of subcutaneous cervical tumors. Moreover, the combination strategy significantly augmented efficacy compared to anti-PD-1 monotherapy by eliciting a robust antitumor immune response.

**Conclusion:**

This study highlights the pivotal role of the STING pathway and the potential of MSA-2 in reshaping the immune microenvironment in cervical cancer. Combining MSA-2 with immune checkpoint inhibitors presents a transformative approach, holding promise for improved prognosis. Further investigations are warranted to explore the broader immune landscape and potential long-term effects of MSA-2 in cervical cancer treatment.

## 1 Background

Cervical cancer poses a substantial global public health challenge for women, as it ranks as the fourth most prevalent gynecological tumor, making it a significant health concern for women on a global scale (1). In the year 2020, an estimated 604,000 new cases of cervical cancer were identified, leading to 342,000 fatalities globally as a consequence of this malignant condition (2). This unsettling statistic underscores the urgent need for comprehensive strategies to address this pervasive and often fatal disease. Notably, over 95% of cervical cancer cases are attributed to persistent high-risk human papillomavirus (HPV) infection (3). Despite the existence of prophylactic vaccines targeting carcinogenic HPV types, their efficacy has been somewhat limited. On one hand, women with persistent existing HPV infections remain susceptible to the development of cervical cancer in the later stages of their lives. On the other hand, cervical cancer patients face a grim prognosis due to local invasion and distant lymphatic metastasis (4). Moreover, contemporary therapeutic strategies that encompass a triad of established interventions, namely radiotherapy, chemotherapy, and surgical resection, are encumbered by adverse effects and exhibit limited effectiveness when confronting advanced stages of the disease (5). Therefore, therapeutic alternatives are scarce when dealing with recurrent or metastatic cases.

As cervical cancer advances, it establishes an immunosuppressive environment, compromising the body’s natural defenses. Addressing this challenge is crucial for both prevention and therapy. The integration of human papillomavirus (HPV) in cervical cancer leads to a complex interplay, intertwining immunosuppressive phenotypes and compromised immunosurveillance (6). Within this landscape, immunotherapy has emerged as a beacon of promise, as underscored by the recent approval of a programmed cell death protein 1 (PD-1)-blocking antibody for the management of recurrent or metastatic disease (7). Remarkably, the US Food and Drug Administration (FDA) approval of pembrolizumab for recurrent and advanced-stage cervical cancer in 2021 (8). Besides, the European Medicine Agency (EMA) approved Cemiplimab, a PD-1 specific antibody, in 2022 due to its significant enhancement in overall survival of cervical cancer (12.0 versus 8.5 months, HR = 0.69) (9). Ongoing clinical trials explore additional immunotherapies, signaling a potential paradigm shift in cervical cancer treatment by enhancing innate antitumor effects and reactivating the immune system (10).

The majority of patients with cervical cancer, however, exhibit limited response to immune checkpoint inhibitors (ICBs) due to diverse mechanisms, thereby significantly thwarting the efficacy of the therapeutic regimen (11). The intricate interplay of specific immune cells, cytokines, and chemokines paints a vivid portrait of the multifaceted tumor microenvironment (TME), each facet characterized by distinct immune phenotypes: the immune inflammation type, the immune excluded type, and the immune desert type (12, 13). Within the heterogeneous expanse of tumor cells, a complex tapestry emerges, often characterized by the simultaneous presence of three distinct TME profiles (14). This intricate landscape is particularly conspicuous in the context of refractory, metastatic, and advanced cancer stages, where the TME frequently assumes the disheartening guise of an immune excluded and desert conditions (15). The utilization of ICB notwithstanding, the clinical objective response rate (ORR) to immune checkpoint inhibitors remains scarcely more than 20% in practical settings (16–20). The integration of treatment strategies has emerged as an unequivocal imperative for effectively addressing the prevailing issue, leading to a pronounced amelioration in prognosis. This holds true whether we are considering combining with conventional chemotherapy or innovative targeting therapeutic regimens (21–23). Especially when combined with other immune-targeted drugs that have the potential to influence various facets of tumor immune recognition, activation, and functional execution, this approach holds the promise of effectively reversing immune excluded and immune desert phenotypes. This synergistic combination enhances the sensitivity of immune checkpoint inhibitors, yielding a more potent therapeutic effect (24–26). These synergies are poised to potentiate the host’s cellular immune response, ultimately directing its focus toward the eradication of HPV-positive cancer cells (27). In this context, it is imperative to recognize the pivotal role of STING, a transmembrane protein that orchestrates a robust immune response by detecting foreign invaders and danger signals. It activates immune cells through a cascade of events and bridges innate and adaptive immune responses, making it a significant therapeutic target (28, 29). CCL5, and CXCL10 has been identified as the downregulation of STING/IFN signaling activated and ulteriorly recruit lymphoid effect immune cells (30, 31). While, CXCL9 exerts as a crucial downstream factor in STING pathway of myeloid effect cells (32). In an *in vitro* experiment, the suppression of STING expression has been shown to diminish the viability of cervical cancer cells. Additionally, findings from *in vivo* investigations highlight that the activation of STING leads to a substantial increase in the populations of CD8+ T cells and CD103+ dendritic cells (DCs) (33). However, the intra-tumoral approach of drug delivery seems a setback for its clinical utilization. A groundbreaking innovation has emerged with the advent of MSA-2, an oral STING agonist, which addresses the critical once consistent existent flaw, ushering in a new era of clinical possibilities (34). Furthermore, there is a dearth of research regarding the implementation of MSA2 in the treatment of cervical cancer. Furthermore, there has been a conspicuous absence of studies exploring the synergistic potential of combining a STING agonist with ICB in the specific context of cervical cancer.

## 2 Materials and methods

### 2.1 Data available source

The expression profiles and clinicopathological parameters were obtained from The Cancer Genome Atlas (TCGA), which can be accessed for download through the UCSC website (https://xenabrowser.net/). Additionally, we utilized the Gene Expression Omnibus (GEO) dataset GSE192897 (35). Relevant web addresses for online resources and analysis tools are thoughtfully provided within the context for easy access.

### 2.2 Prognostic analysis and immune infiltration analysis

The prognostic analysis of STING downstream genes, which include CCL5, CXCL9, and CXCL10 in cervical cancer, is graciously facilitated by the open-access platform, Kaplan-Meier Plotter (36). Additionally, the expression of these STING downstream genes was stratified based on the clinical stage of cervical cancer patients.

### 2.3 Immune infiltration and immune cell analysis

We performed a log_2_(x+0.001) transformation of the expression values of STING downstream genes for each sample in the TCGA-CESC database. Additionally, we extracted gene expression profiles for each tumor and mapped them to GeneSymbol. Furthermore, we utilized the R package ESTIMATE and *deconvo_CIBERSOR* method from the R package IOBR to reevaluate the immune infiltration score and immune cell infiltration in each patient with cervical cancer based on gene expression (37–39). Ultimately, We calculated the Pearson’s correlation coefficient between genes and the aforementioned immune infiltration states in various tumors using the *corr.test* function from the R software package psych (version 2.1.6). All the data about this section were obtained from TCGA and analyses were processed through the online platform, http://sangerbox.com/home.html (40).

### 2.4 Single-cell RNA-seq (scRNA-seq) analysis

In this study, we analyzed the alterations in the TME after MSA-2 treatment in an immune-excluded tumor model (26). Our analytical journey commenced with the intricate task of annotating cell types using the SingleR package (version 1.8.0) paired with the authoritative ImmGen reference database. To fortify the credibility of our annotations obtained via SingleR, we ventured further by meticulously calculating the expression levels of immune cell-specific markers, drawing from a compendium of previous studies. In our pursuit of accuracy, T cell subclusters were annotated concerning well-established markers, while NK cell subclusters were meticulously defined based on the robust criteria of Itgam and CD27 expression levels. Subsequently, we employed the venerable chi-squared test to scrutinize disparities in cluster proportions among various experimental groups. This statistical examination offered valuable insights into the differences observed. Our visualization and characterization efforts were no less meticulous. We artistically portrayed the distinctive features of T cells, NK cells, and conventional dendritic cells (cDCs) across different experimental groups, invoking the well-respected MSigDB hallmark gene sets (H). The signaling enrichment was assessed through the judicious use of the Gene Set Enrichment Analysis (GSEA), facilitated by the singleseqgset package (version 1.2.9) (26). The evaluation of intercellular communication numbers and intensities utilized the CellChat package (version 1.6.1) with the CellChatDB.mouse database. Visualization of cell communication was accomplished through the *netVisual*, *netVisual_heatmap*, the *netAnalysis_signalingChanges_scatter*, and *netVisual_bubble* functions.

### 2.5 Cell lines culture and therapeutic agents

Murine cervical cancer cell lines U14, TC-1 were cultured in Dulbecco’s Modified Eagle’s Medium (DMEM) (Gibco) with 10% fetal bovine serum (Gibco) and 1×Penicillin-Streptomycin Solution (Gibco). Murine PD-1 antibody (29F.1A12) was purchased from BioXCell. MSA-2 (HY-136927) was purchased from MedChemExpress (MCE).

### 2.6 Murine tumor models

We conducted an investigation into the antitumor activity of the combination of MSA-2 and anti-PD-1 in two syngeneic tumor models. U14 and TC-1 are commonly used cervical tumor models to explore immunotherapy efficacy (41, 42). MSA-2 was administered orally at a single dose of 50 mg/kg, while mice received 5 mg/kg of anti-PD-1 treatment on alternate days, for a total of three treatments. Tumor volume was assessed every other day, and mice were euthanized either when their tumor volume exceeded 2000 mm^3^ or at the termination of the experiment. In the case of subcutaneous tumor models, we initially implanted 5 × 10^6^ U14 or TC-1 cells into the subcutaneous tissue of C57BL/6 mice. All mice carrying tumors were randomly divided into four groups: the control group (CTL), the MSA-2 group, the anti-PD-1 group, and the combination therapy group with MSA-2 and anti-PD-1 (each group consisting of 5 mice). Treatment commenced 10 days after injection into the subcutis or when the tumor volume reached a range of 50 ~ 150 mm^3^. We repeated the U14 model to investigate the impact of agents on survival, with an initial implantation cell count of 7.5 × 10^6^.

### 2.7 Immunofluorescent (IF) staining assay

Tissues were fixed in a 4% paraformaldehyde solution overnight. After fixation, the tissues underwent three PBS rinses. Subsequently, they were immersed in ethyl alcohol with an escalating concentration for one hour each. Following this, the tissues underwent dehydration and were then embedded in paraffin wax at 65°C, ultimately facilitating slicing for further analysis. IF staining was conducted using the tyramide signal amplification method. Antibodies targeting PCNA (nucleic staining, BM0104, Boster), anti-CD3 (ab231775, Abcam), and anti-CD8 (ab217344, Abcam) were employed in the assays. The Tunel assay (C1086, Beyotime) was performed following the manufacturer’s recommendations. Captured images were examined using Caseviewer or Hamamatsu Nanozoomer software, and two independent pathologists delineated the regions of interest (ROIs). The integral optical density (IOD) was quantified by Image-Pro Plus 6.0 (Media Cybernetics) used to measure immune cell infiltration status and apoptotic tumor cells.

### 2.8 Data statistical analyses

Statistical analyses were conducted using both GraphPad Prism (version 8.4.2) and R software. Student’s t-test was employed to compare two variables when the data followed a normal distribution. All statistical analyses in this study were two-sided, and statistical significance was defined as p < 0.05.

## 3 Results

### 3.1 The prognosis of cervical cancer is vigorously correlated with the expression of STING downstream genes

**Figure 1:**
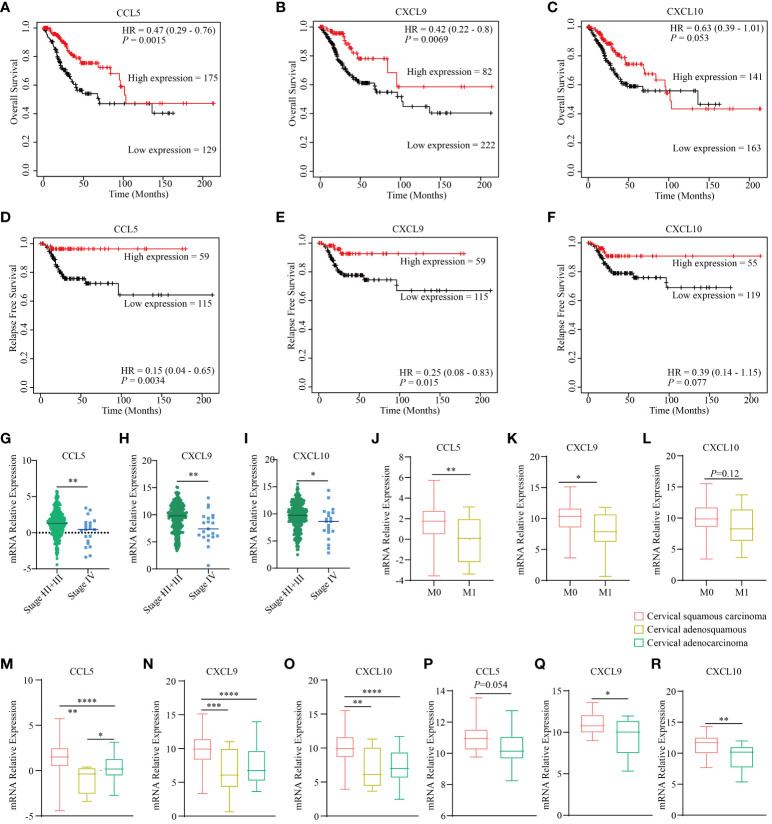
STING downstream genes are correlated with the clinical outcomes and traits of cervical cancer. The curves illustrate the overall survival of cervical cancer patients stratified based on the expression status of three downstream genes of STING, namely CCL5 **(A)**, CXCL9 **(B)**, and CXCL10 **(C)** based on the TCGA dataset. The curves present the relapse free survival of patients with cervical cancer grouped by the expression level of three downstream genes of STING, namely CCL5 **(D)**, CXCL9 **(E)**, and CXCL10 **(F)** according to TCGA dataset. The diagram depicts the clinical stages of different expression levels of CCL5 **(G)**, CXCL9 **(H)**, and CXCL10 **(I)** based on TCGA dataset. The diagram depicts the clinical metastatic status of different expression levels of CCL5 **(J)**, CXCL9 **(K)**, and CXCL10 **(L)** based on TCGA dataset. The expression level of CCL5 **(M)**, CXCL9 **(N)**, and CXCL10 **(O)** in different histological types based on TCGA dataset. The expression level of CCL5 **(P)**, CXCL9 **(Q)**, and CXCL10 **(R)** in different histological types according to the GSE192897 dataset. * in this figure represents *P* < 0.05, ** represents *P* < 0.01, *** indicates *P* < 0.001, and **** represents *P* < 0.0001.

Previous investigations have identified CCL5, CXCL9, and CXCL10 as downstream targets of STING, which serve as crucial intermediaries connecting DNA sensing with the initiation of the immune response (43, 44). Notably, we uncovered a compelling association between CCL5, CXCL9, and CXCL10 and key prognostic factors, encompassing overall survival and progression-free survival, in cervical cancer, as observed within TCGA dataset. The conspicuous elevation in expression levels of CCL5, CXCL9, and CXCL10 consistently serves as a robust prognostic indicator for prolonged survival and significantly reduced probabilities of recurrence and relapse (**Figures 1A–F**). Moreover, in the context of advanced stages of cervical cancer, there is a noticeable decrease in the expression of these genes (**Figures 1G–I**), and they are closely linked to a conspicuous inverse correlation with metastatic potential (**Figures 1J–L**). Additionally, they present a diminished expression profile in cervical adenocarcinoma compared to squamous cervical carcinoma both in TCGA dataset (**Figures 1M–O**) and the GSE192897 dataset (**Figures 1P–R**). Previous studies demonstrated that cervical adenocarcinoma possessed a heightened malignant propensity, and a greater inclination toward metastatic behavior (45), characterized by the TME with a more robust immune infiltration cells profile (46). These findings underscore the pivotal role of the STING pathway in shaping the prognosis of cervical cancer.

### 3.2 The STING downstream genes are correlated with the immune infiltration in the TME of cervical cancer

**Figure 2:**
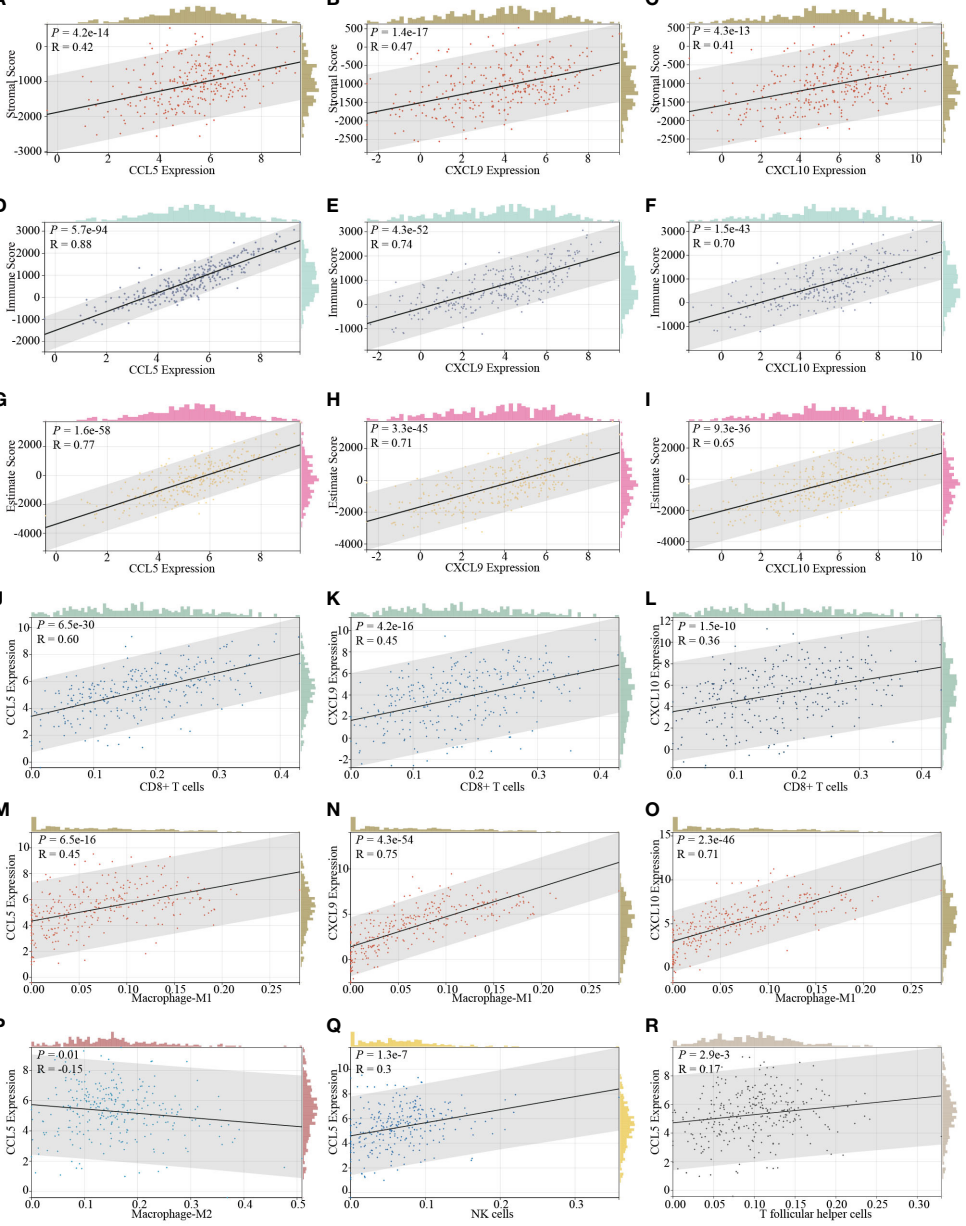
The correlation of STING downstream genes and immune infiltration characteristics in cervical cancer. The expression of CCL5 **(A)**, CXCL9 **(B)**, and CXCL10 **(C)** is positively relative to the stromal score determined by the ESTIMATE SCORING assessing system. The expression of CCL5 **(D)**, CXCL9 **(E)**, and CXCL10 **(F)** positively correlated with the immune score determined by the ESTIMATE SCORING assessing system. The expression of CCL5 **(G)**, CXCL9 **(H)**, and CXCL10 **(I)** exhibits a positive correlation with the estimate score determined by the ESTIMATE SCORING assessing system. The expression of CCL5 **(J)**, CXCL9 **(K)**, and CXCL10 **(L)** is positively correlated with the infiltration of CD8+ T cells. The expression of CCL5 **(M)**, CXCL9 **(N)**, and CXCL10 **(O)** is positively correlative with the infiltration of M1 type macrophages. The expression of CCL5 is additionally negatively correlated with the infiltration of M2 type macrophages **(P)** and positively correlated with the infiltration of NK cells **(Q)**, and T follicular helper cells **(R)**.

As previously implied, it becomes increasingly evident that various histotypes of cervical cancer exhibit unique and discernible immune infiltration cell profiles. This nuanced variation, in turn, exerts a profound influence on the dynamic landscape of tumor invasiveness and critically impacts the efficacy of drug treatments. With this in mind, our subsequent investigation delves into the intricate correlation between the expression levels of STING downstream genes, CCL5, CXCL9, and CXCL10, and their influence on the overall immune infiltration status within the cervical cancer TME. After analysis, we determine that these genes are all vigorously positively correlated to three dimensions of immune infiltration, namely stromal scores (**Figures 2A–C**), immune scores (**Figures 2D–F**), and estimate scores (**Figures 2G–I**) based on ESTIMATE comprehensive scoring in the context of cervical cancer. Besides, When delving deeper into specific immune cells, we identify consistency of the expression of all these genes and the presence of CD8^+^ T cells (**Figures 2J–L**) and M1-type macrophages (**Figures 2M–O**). Moreover, CCL5 expression, in particular, demonstrates an inverse relationship with M2-type macrophages (**Figure 2P**) and a direct association with NK cells (**Figure 2Q**) and T follicular helper cells (**Figure 2R**). It is firmly established that CD8+ T cells, M1-type macrophages, NK cells, and T follicular helper cells not only serve as instrumental agents in the immune clearance of tumor cells but also serve as indicative barometers of the potential efficacy of ICB therapy (47–52). Conversely, M2-type macrophages exhibit contrasting effects (49).

### 3.3 scRNA-seq uncovers the immune landscape when STING agonist is utilized in an immune-excluded model

**Figure 3:**
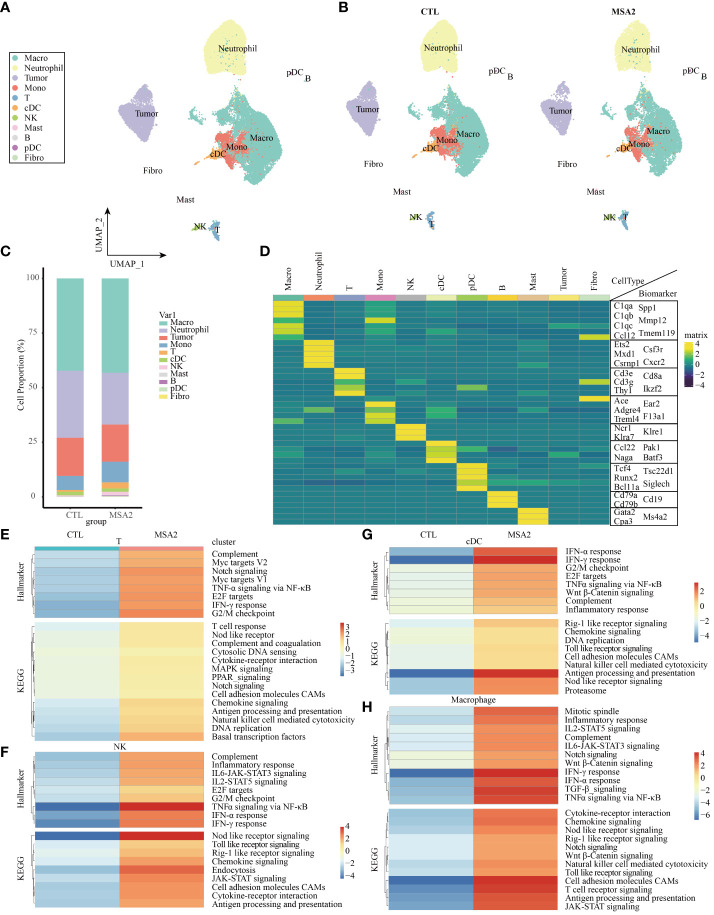
The scRNA-seq analyses demonstrated the comprehensive immune landscape variations of TME after MSA-2 administration. **(A)** The cell clustering distribution of TME through secondary scRNA-seq analysis. **(B)** The alternations of cell distributions in TME of a breast cold tumor model between the control group (CTL) and MSA-2 treatment group. **(C)** The detailed cell proportions between CTL and MSA-2 treatment group. **(D)** The specific biomarkers of each component within TME. The signaling variations of T cells **(E)**, NK cells **(F)**, cDCs **(G)**, and macrophages **(H)**.

In our quest for an elaborate comprehension of the nuanced alterations of diverse immune cell subsets in the realm of MSA-2 therapy, we embarked on a secondary analysis within an immune-excluded tumor model (EMT-6), encompassing an array of immune system components (**Figure 3A**). Our initial focus was on the exploration of functional disparities between the MSA-2 treatment group and the CTL group within the broader population of TME cells (**Figure 3B**) (26). Notably, the MSA-2 group exhibited a substantially higher proportion of T cells and NK cells compared to the CTL group, while the proportion of tumor cells exhibited a converse trend (**Figure 3C**). The diverse immune cell populations are distinguished by their specific biomarkers. For example, annotations of *Ncr*1, *Klra7* and *Klree1* designate NK cells, and annotations of *Cd79a*, *Cd79b* and *Cd19* represent B cells (**Figure 3D**). The GSEA analysis is subsequently conducted to explore the inherent discrepancies between the two groups in the context of the immune cell population. After administration of MSA-2, T cells are characterized within the GO annotation by the escalation of the level of Myc target genes, notch signaling associated genes, TNF-α associated genes, E2F target genes, IFN-γ associated genes, and G2/M associated genes (**Figure 3E**). In the scale of KEGG annotation, the levels of immune-activating and response signaling also significantly increase (**Figure 3E**). In the domain of NK cells, as formidable sentinels orchestrating immune surveillance and targeted elimination of tumor cells, the results of GSEA reveal a noteworthy similarity in immune-stimulating signaling between NK cells and T cells after MSA-2 administrated. However, the NK cells signaling profile undergoes the additional influence of IL-6 and IL-2 signals (**Figure 3F**). In the transcriptive profile of cDCs and macrophages, the aforementioned immune-facilitating signal processes are similarly observed, but their specific distinctive activating signaling presents simultaneously (**Figures 3G, H**).

### 3.4 Following the administration of MSA-2, there is an enhancement in the interactions among the components in the TME

**Figure 4:**
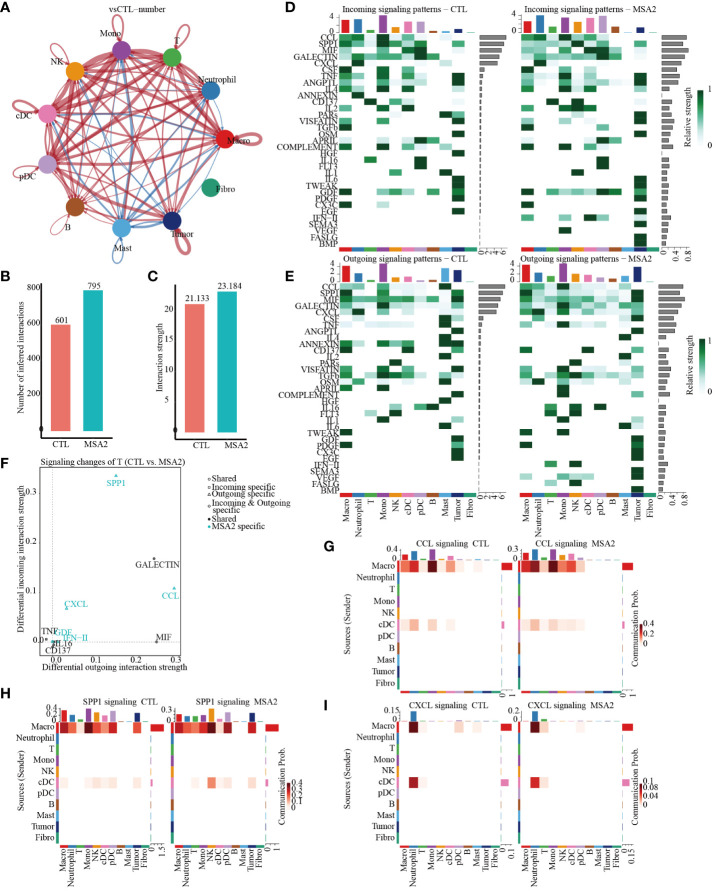
The cellular interactions between components in TME through secondary analyses of scRNA-seq following MSA-2 utilized. **(A)** The size of the circles corresponds to the population size of the cell groups, while the thickness of the edges signifies the intensity of interaction between these populations. The red-colored loops were strengthened in the MSA-2 group, and the blue-colored loops were strengthened in the CTL group. **(B)** The bar plot demonstrates the disparity in quantification of cellular interactions between the CTL and MSA-2 groups. **(C)** Bar plot depicts the discrepancies in the strength of interaction among TME components. **(D)** Heatmaps indicate the strength of incoming signaling patterns of components of the TME. **(E)** Heatmaps indicate the strength of outcoming signaling patterns of components of the TME. **(F)** Volcano plot depicts the most significant signaling in the context of both incoming and outcoming signaling. Heatmaps illustrate the disparities in communication probability among TME cells between the CTL group and MSA2 group in relation to CCL signaling **(G)**, SPP1 signaling **(H)**, and CXCL signaling **(I)**.

**Figure 5:**
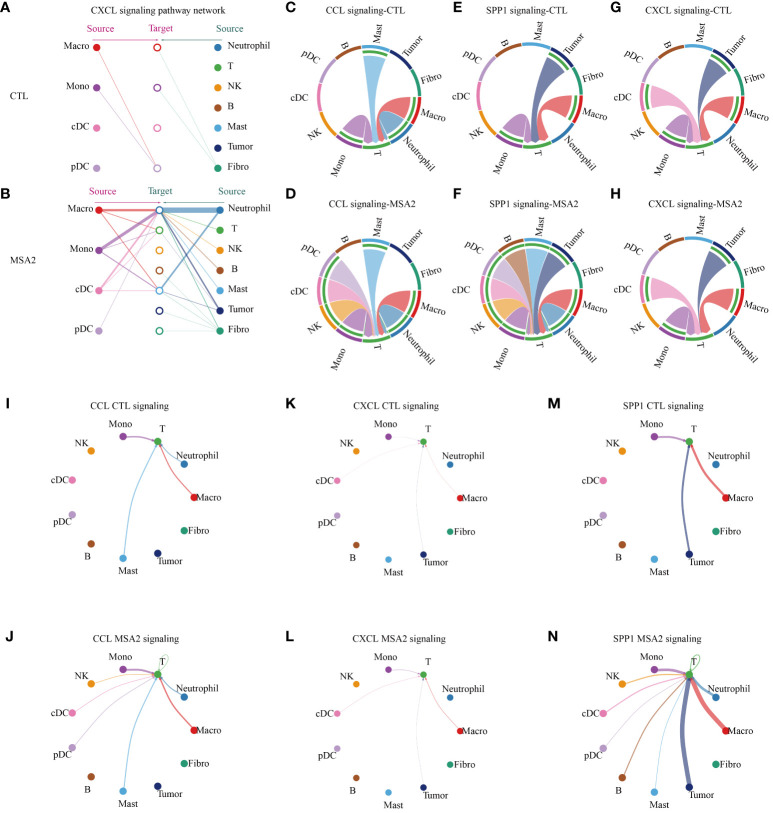
The cellular interactions between components in TME. In the CXCL signaling pattern, the interaction between antigen presenting cells (macrophage, monocytes, cDC and pDC) and other components in TME of CTL group **(A)** or MSA2 group **(B)**. **(C–H)** The plots illuminate, in each intriguing signaling pattern, the intercellular interactions circled T cells. **(I–N)** The plots indicate, in the realms of each interesting signaling, the interaction strength of each cellular communication centered on T cells. The thickness of each line infers the interaction strength.

Then, the CellChat package was utilized to infer biologically significant cell communication events (**Supplementary Files: Supplementary Figures S1**, **S2**). The MSA-2 group, intriguingly, demonstrated a comprehensive enhancement in cellular communications, encompassing both the number and strength of interactions (**Figures 4A–C**). Significantly, MSA-2 augmented intercellular communication within immune response pathways, including C-C motif chemokine ligand (CCL), Secreted Phosphoprotein 1 (SPP1), C-X-C motif chemokine ligand (CXCL), TNF, Growth Differentiation Factor (GDF), and IFN-II (**Figures 4D, E**). Notably, CCL, SPP1, and CXCL were specific to MSA-2 (**Figure 4F**). Subsequently, we conducted a comprehensive examination of cell signaling pathways unique to the MSA-2 and CTL groups across different cellular subtypes (**Figures 4G–I**). Of all the signaling patterns, CXCL exhibits modest cellular interaction but presented a relatively higher correlation with prognosis in our previous analyses. More interaction analysis on CXCL signaling patterns was conducted, and it revealed that there was higher communication strength between antigen presentation cells and other components with the MSA treatment through autocrine and paracrine effects under the CXCL signaling pattern (**Figure 5A**). In T cells, besides biologically significant cell communications were observed involving CCL, SPP1, and CXCL pathways, exclusive to the MSA-2 group. Recognizing the crucial role of T cells in the cancer-immunity circle, intercellular interactions centered around T cells were highlighted within the CCL, SPP1, and CXCL pathways. Within CCL signaling, MSA-2 significantly strengthened communications between T cells and macrophages/monocytes/neutrophils, and improved the communications between T cells and NK cells/cDC/pDC/T cells compared to CTL (**Figures 5C, D, I, J**). In the realm of SPP1 signaling, MSA-2 strikingly consolidated communications between T cells and macrophages/monocytes/tumor cells, particularly tumor cells. Additionally, it amplified intercellular interactions of T cells with NK cells/cDC/pDC/B cells/mast cells/T cells in comparison with CTL (**Figures 5E, F, K, L**). Analogously, CXCL signaling moderately fortified the interactions between T cells and monocytes/cDC/tumor cells/macrophages (**Figures 5G, H, M, N**).

### 3.5 MSA-2, both in isolation and in combination with PD-1 inhibitor suppressed the subcutaneous cervical tumor growth, with immensely stimulating antitumor immune response

**Figure 6:**
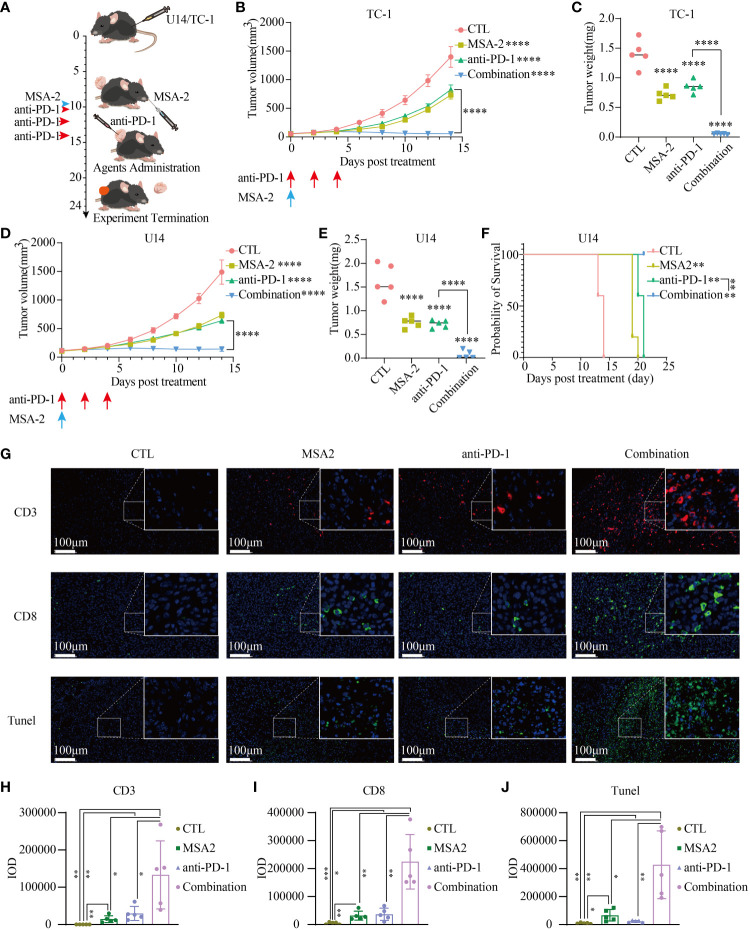
MSA-2 synergized anti-PD-1 in antitumor immunity. **(A)** The schematic diagram of *in vivo* murine experiment of a combination of MSA-2 and anti-PD-1 treatment. The first day began with the subcutaneous tumor cells’ transplantation by the groin of the mouse. The tumor volume was measured and the living condition was assessed every two days until the tenth day as time elapsed. On the tenth day, MSA-2 was administered orally, while anti-PD-1 was intravenously injected. In the combination group and anti-PD-1 group, anti-PD-1 was subsequently injected twice additionally. The tumor volume and living conditions were continuously undertaken every two days. By the termination of the experiment, the subcutaneous tumor was harvested and underwent further processing. The curves represent the variation tendency of subcutaneous tumor of TC-1 **(B)** and U14 **(D)** respectively using the algorithm of “major axis × minor axis × minor axis × 0.5”. At the termination of the experiment, the mice were euthanatized and the tumors were cut off and weighed. The weight of the tumor from each group was recorded and exhibited in the plot (**C** representing the TC-1 group and **E** representing the U14 group). **(F)** The overall survival curve of U14 C54BL/6 model. **(G)** IF of the tissue from the U14 group showed the CD3+, CD8+ and Tunel staining of each group. **(H–J)** IOD of each IF staining was evaluated. * in this figure indicates *P* < 0.05, ** indicates *P* < 0.01, *** indicates *P* < 0.001, and **** represents *P* < 0.0001.

To assess the feasibility of using MSA-2 as a monotherapy or as a synergistic agent with ICB in cervical cancer treatment, we then conducted an *in vivo* murine assay (**Figure 6A**). After the administration of MSA-2, the tumor volume was significantly reduced in mice, whether they were treated with anti-PD-1 or not, as compared to the CTL group (**Figures 6B, D**). Furthermore, the combination treatment of MSA-2 and anti-PD-1 exhibited a remarkably suppressive effect on tumor growth when compared to either MSA-2 administration or anti-PD-1 monotherapy (**Figures 6B, D**). Upon termination of the experiment, following euthanasia, the weight of each tumor was assessed, confirming the previous observation that MSA-2 administration effectively suppressed tumor growth and synergistically enhanced the therapeutic efficacy of anti-PD-1 treatment (**Figures 6C, E**). The overall survival of C57BL/6 mice with subcutaneous U14 cervical cancer cells was significantly prolonged by both MSA-2 and anti-PD-1 monotherapy. Moreover, the combination strategy of MSA-2 and anti-PD-1 demonstrated a remarkable extension in overall survival of U14 C57BL/6 models, surpassing the efficacy observed with anti-PD-1 monotherapy (**Figure 6F**). The IF analysis of murine U14 tumor tissue was subsequently conducted, revealing a more extensive distribution of CD3+ and CD8+ cells in the combination groups compared to the anti-PD-1, MSA-2, or CTL group (**Figures 6G–I**). Meanwhile, the TUNEL assay was performed to assess the apoptotic status of tumor tissue, illuminating a higher abundance of apoptotic tumor cells in the synergistic therapeutic approach involving MSA-2 and anti-PD-1 (**Figures 6G, J**).

## 4 Discussions

Cervical cancer poses a significant global public health challenge, ranking as the fourth most prevalent gynecological tumor and contributing substantially to women’s health concerns worldwide. Current therapeutic strategies, including radiotherapy, chemotherapy, and surgical resection, face challenges due to adverse effects and limited effectiveness in advanced stages of the disease. As cervical cancer progresses, it creates an immunosuppressive environment, weakening the body’s natural defenses against cancer. In light of these challenges, exploring new perspectives for both prevention and therapeutic management becomes imperative. The integration of immunotherapy involved in cancer treatment regimens has emerged as promising, exemplified by the recent approval of PD-1 blocking antibodies for recurrent or metastatic cervical cancer. However, challenges persist in achieving significant responses to ICBs, with clinical response rates remaining modest, which is contributed by TME complexity, and characterized by distinct immune phenotypes. Strategies combining ICB with conventional chemotherapy or innovative targeting regimens have emerged as imperative for addressing this issue. The advent of MSA-2, an oral STING agonist, represents a groundbreaking innovation, addressing the limitations of intra-tumoral drug delivery and opening new clinical possibilities. Under these considerations, we conducted a study that delves into the prognostic significance of STING downstream genes in cervical cancer, establishing their correlation with overall survival, progression-free survival, and immune infiltration status. As previously acknowledged, CCL5 and CXCL10 as pivotal signaling bridges between NK cells and T cells, and tumor cells (31). CXCL9 in myeloid cell triggered by STING activated serves as an intermediatory agent invigorating the secretion of IFNγ in T cells that reciprocally enhances the expression of CXCL9 in myeloid cells (32). between The downstream genes CCL5, CXCL9, and CXCL10 exhibit elevated expression levels associated with prolonged survival and reduced recurrence probabilities. The CCL5, CXCL9, and CXCL10 corporately reflect the activity of STING pathway. The positive correlation with immune infiltration further underscores the critical role of the STING pathway in shaping the prognosis of cervical cancer. The exploration of immune infiltration in the TME reveals a positive correlation between STING downstream genes and stromal scores, immune scores, and estimate scores, suggesting their influence on the overall immune landscape in cervical cancer. The association with specific immune cell populations, such as CD8^+^ T cells, M1-type macrophages, NK cells, and T follicular helper cells, provides insights into STING upregulation as a potential target for immunotherapeutic interventions. Therefore, the novel oral STING agonism, MSA-2 was presumed as an immune stimulus in cervical cancer. To verify the hypothesis, single-cell RNA-seq analysis in a breast cold tumor model elucidates the impact of MSA-2 on diverse immune cell subsets. The gene expression profile revealed upregulation of several certified immune-activated gene clusters, including TNF-α and IFN-γ, in tumor immunity effect cells following MSA administration (53–55). The higher proportion of T cells and NK cells, coupled with signaling enrichment in immune-activating pathways, and intensifying the intercellular interactions among the components within TME, highlights the potential of MSA-2 in modulating the immune landscape. The *in vivo* murine assay further demonstrates the efficacy of MSA-2, both as a standalone treatment and in combination with anti-PD-1, in effectively suppressing subcutaneous cervical tumor growth. Additionally, it stimulates a robust antitumor immune response that ultimately leads to tumor cell apoptosis. However, further investigations are warranted to comprehensively characterize the immune microenvironment of tumor-infiltrating immune cells in cervical cancer following MSA-2 functional modulation, beyond mere quantitive assessment of CD8+ T cells through IF assays. Besides, the absence of long-term immune-related toxicities or autoimmunity in mice following MSA-2 administration was attributed to experimental temporal restrictions, necessitating further evaluation.

## 5 Conclusions

In summary, this thorough investigation traverses the complex terrain of cervical cancer, highlighting the critical function of the STING pathway and the significant promise of MSA-2 in altering the immune microenvironment. The results emphasize the necessity of integrating immunotherapies, especially the synergistic interaction between MSA-2 and ICBs, as a revolutionary method in the management of cervical cancer. Future research endeavors should concentrate on conducting clinical trials involving MSA-2 and ICBs, determining biomarkers that predict responses to therapy, and investigating the efficacy of combined therapeutic strategies. Such initiatives are crucial for the application of these findings to enhance patient outcomes. Moving forward, the insights gained from this study lay the groundwork for novel treatment approaches, offering hope for the improvement of prognosis in patients with cervical cancer.

## Data availability statement

The datasets presented in this study can be found in online repositories. The names of the repository/repositories and accession number(s) can be found in the article/**Supplementary Material**. The raw data of single-cell RNA sequence was from a tumor-excluded model (EMT-6) from a previously published article (26).

## Ethics statement

Ethical approval was not required for the study involving humans in accordance with the local legislation and institutional requirements. Written informed consent to participate in this study was not required from the participants or the participants’ legal guardians/next of kin in accordance with the national legislation and the institutional requirements. The animal study was approved by Animal Ethics Committee, the Second Affiliated Hospital of Zhejiang University School of Medicine. The study was conducted in accordance with the local legislation and institutional requirements.

## Author contributions

JZ: Investigation, Writing – review & editing, Conceptualization, Funding acquisition, Supervision. TL: Project administration, Validation, Writing – review & editing, Data curation, Formal Analysis, Investigation, Methodology, Resources, Software, Visualization, Writing – original draft. WZ: Writing – review & editing, Project administration, Supervision, Validation. MN: Methodology, Writing – review & editing. YW: Writing – review & editing, Methodology. XD: Conceptualization, Funding acquisition, Supervision, Writing – review & editing, Project administration, Validation.

## Supplementary material

Supplementary Figure 1
**(A)** The total amount of intercellular interactions among the various components in the TME. **(B)** The weight or strength of total interactions among the multiple components within the TME. **(C)** The weight or strength of the interactions initiated by T cells with all the components in the TME.

Supplementary Figure 2 The extent of intercellular communications between each component within TME.
