# Supplementary material for: STING agonist inflames the cervical cancer immune microenvironment and overcomes anti-PD-1 therapy resistance
Source: Front Immunol. 2024 Mar 14;15:1342647. doi: 10.3389/fimmu.2024.1342647 (PMC10972971; doi:10.3389/fimmu.2024.1342647)
Supplement: Supplementary file 1 [file Table_1.docx]

The raw data could be obtained from: https://www.jianguoyun.com/p/De3wGHAQy6-WDBj35KkFIAA
