# Supplementary figures and images for: STING agonist inflames the cervical cancer immune microenvironment and overcomes anti-PD-1 therapy resistance
Source: Front Immunol. 2024 Mar 14;15:1342647. doi: 10.3389/fimmu.2024.1342647 (PMC10972971; doi:10.3389/fimmu.2024.1342647)

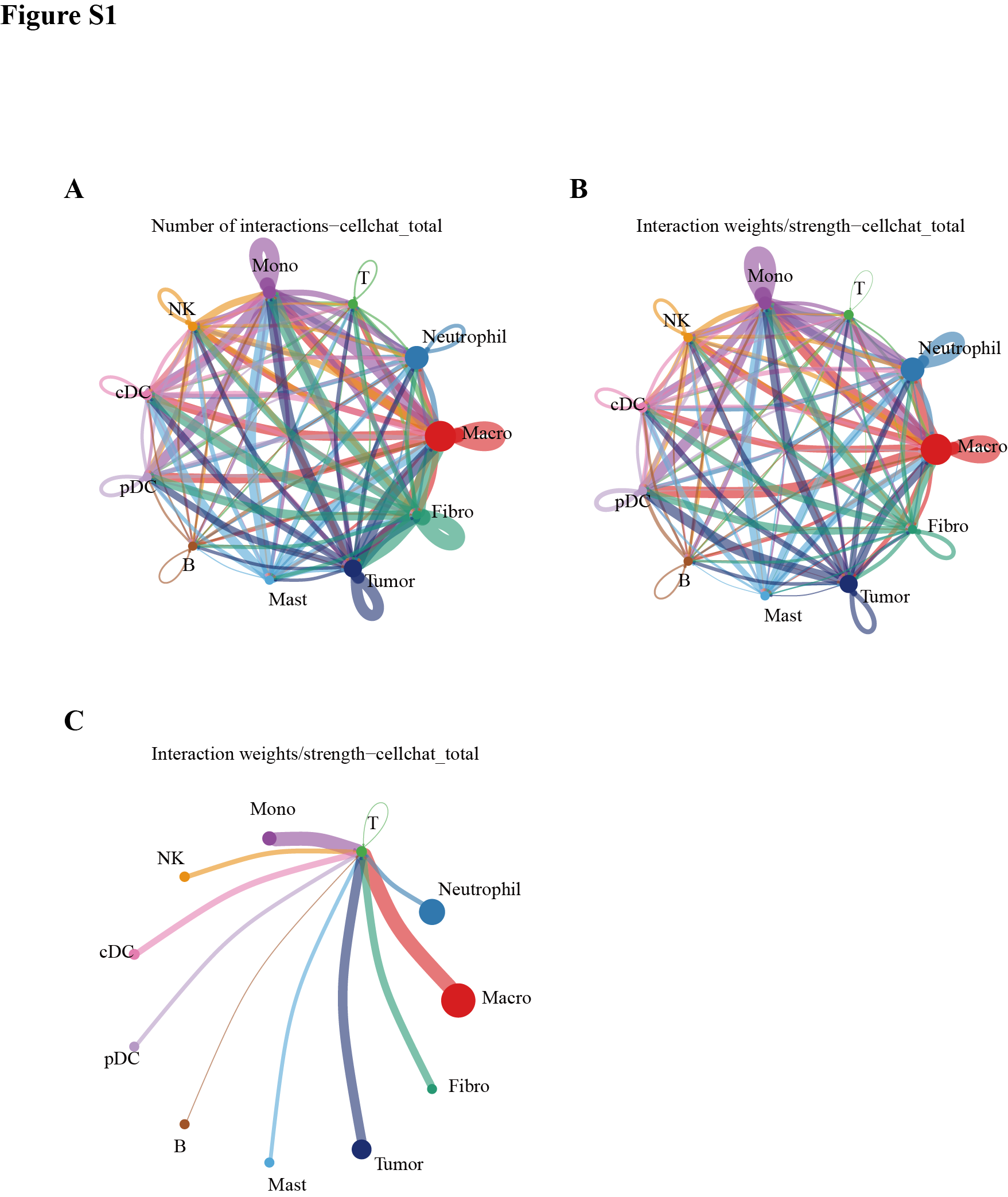

Supplement: Supplementary Figure 1 — (A) The total amount of intercellular interactions among the various components in the TME. (B) The weight or strength of total interactions among the multiple components within the TME. (C) The weight or strength of the interactions initiated by T cells with all the components in the TME. [file Image_1.tif]

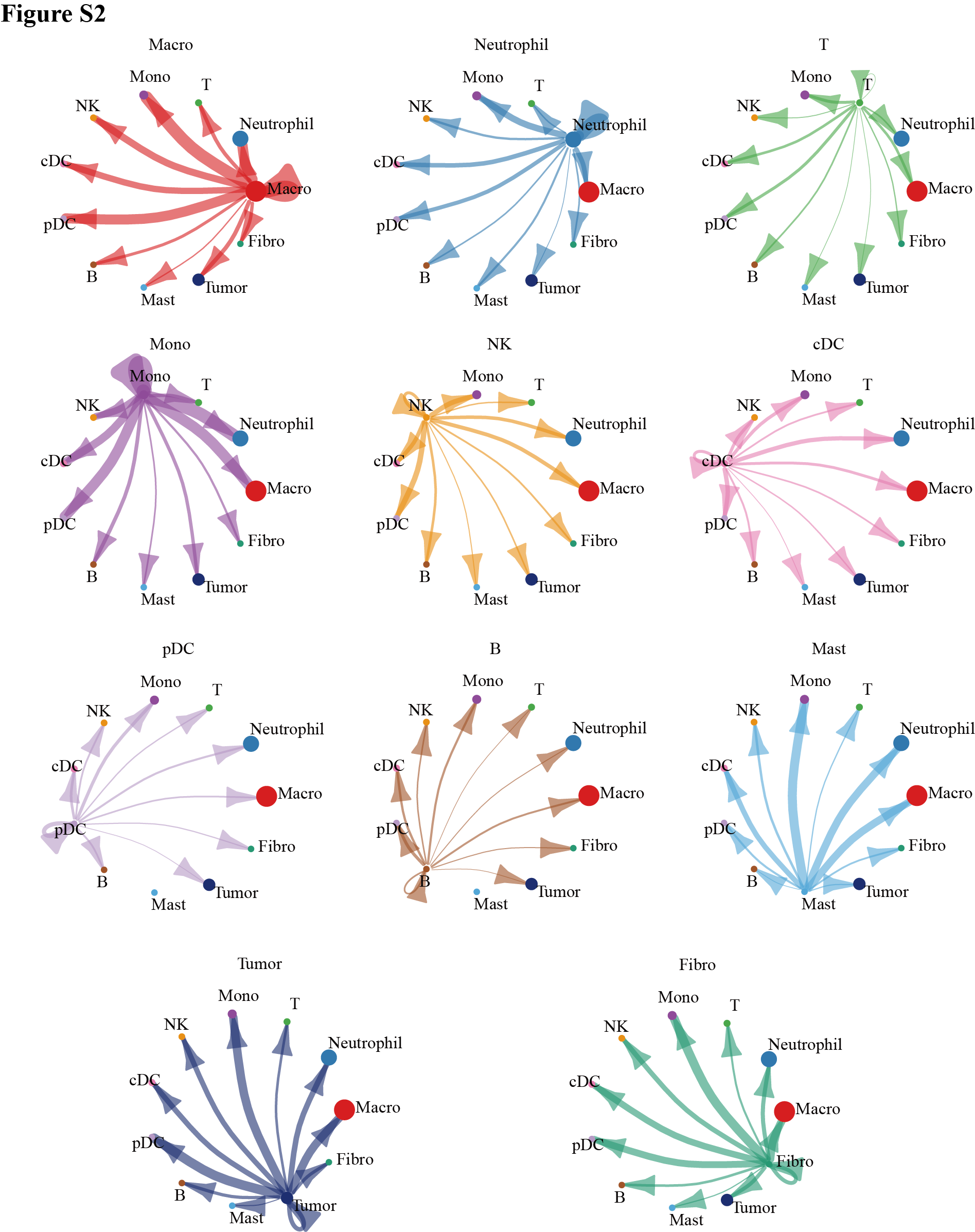

Supplement: Supplementary Figure 2 — The extent of intercellular communications between each component within TME. [file Image_2.tif]
